# Supplementary material for: Multi‐dataset identification of innovative feature genes and molecular mechanisms in keratoconus
Source: J Cell Mol Med. 2024 Sep 19;28(18):e70079. doi: 10.1111/jcmm.70079 (PMC11412914; doi:10.1111/jcmm.70079)
Supplement: Supplementary file 2 — Material S2. [file JCMM-28-e70079-s002.zip › 7.GSEA analysis/3.DBNDD1/KEGG_ETHER_LIPID_METABOLISM.html]

Details for gene set KEGG\_ETHER\_LIPID\_METABOLISM[GSEA]

|  || Dataset | DBNDD1.group.cls#h\_versus\_l |
| Phenotype | group.cls#h\_versus\_l |
| Upregulated in class | l |
| GeneSet | KEGG\_ETHER\_LIPID\_METABOLISM |
| Enrichment Score (ES) | -0.45682535 |
| Normalized Enrichment Score (NES) | -1.1705216 |
| Nominal p-value | 0.275 |
| FDR q-value | 0.70320565 |
| FWER p-Value | 0.993 |
Table: GSEA Results Summary

  

Fig 1: Enrichment plot: KEGG\_ETHER\_LIPID\_METABOLISM      
 Profile of the Running ES Score & Positions of GeneSet Members on the Rank Ordered List

  

| SYMBOL | TITLE | RANK IN GENE LIST | RANK METRIC SCORE | RUNNING ES | CORE ENRICHMENT || 1 | PLA2G2F | na | 6705 | 0.131 | -0.1410 | No |
| 2 | PLA2G10 | na | 7003 | 0.124 | -0.1001 | No |
| 3 | PLA2G2D | na | 8134 | 0.101 | -0.0926 | No |
| 4 | PLA2G12B | na | 9010 | 0.085 | -0.0838 | No |
| 5 | PLA2G7 | na | 11957 | 0.047 | -0.1498 | No |
| 6 | PAFAH2 | na | 13156 | 0.037 | -0.1696 | No |
| 7 | PLA2G6 | na | 14048 | 0.030 | -0.1835 | No |
| 8 | PLD1 | na | 15421 | 0.020 | -0.2152 | No |
| 9 | PLA2G4A | na | 15676 | 0.018 | -0.2153 | No |
| 10 | PAFAH1B3 | na | 16589 | 0.012 | -0.2368 | No |
| 11 | AGPS | na | 16688 | 0.011 | -0.2351 | No |
| 12 | PLA2G2E | na | 17656 | 0.005 | -0.2609 | No |
| 13 | PAFAH1B1 | na | 17848 | 0.004 | -0.2649 | No |
| 14 | PLA2G12A | na | 18106 | 0.002 | -0.2715 | No |
| 15 | PLA2G4E | na | 19468 | -0.008 | -0.3075 | No |
| 16 | PLD2 | na | 20094 | -0.014 | -0.3200 | No |
| 17 | LPCAT4 | na | 21565 | -0.028 | -0.3513 | No |
| 18 | ENPP2 | na | 21942 | -0.032 | -0.3496 | No |
| 19 | PLA2G2C | na | 22365 | -0.036 | -0.3476 | No |
| 20 | LPCAT2 | na | 23073 | -0.044 | -0.3505 | No |
| 21 | JMJD7-PLA2G4B | na | 23109 | -0.044 | -0.3338 | No |
| 22 | CHPT1 | na | 27379 | -0.116 | -0.4106 | Yes |
| 23 | PAFAH1B2 | na | 27743 | -0.125 | -0.3713 | Yes |
| 24 | ENPP6 | na | 28130 | -0.134 | -0.3291 | Yes |
| 25 | PLA2G3 | na | 28607 | -0.146 | -0.2845 | Yes |
| 26 | PLA2G5 | na | 29074 | -0.159 | -0.2347 | Yes |
| 27 | LPCAT1 | na | 31025 | -0.221 | -0.2028 | Yes |
| 28 | PLA2G1B | na | 31847 | -0.256 | -0.1246 | Yes |
| 29 | PLA2G2A | na | 34424 | -0.522 | 0.0088 | Yes |
Table: GSEA details [plain text format]

  

Fig 2: KEGG\_ETHER\_LIPID\_METABOLISM      
 Blue-Pink O' Gram in the Space of the Analyzed GeneSet

  

Fig 3: KEGG\_ETHER\_LIPID\_METABOLISM: Random ES distribution      
 Gene set null distribution of ES for **KEGG\_ETHER\_LIPID\_METABOLISM**
